# Mechanism of injury, injury patterns and associated injuries in patients operated for chest wall trauma

**DOI:** 10.1007/s00068-019-01119-z

**Published:** 2019-04-06

**Authors:** Eva-Corina Caragounis, Yao Xiao, Hans Granhed

**Affiliations:** Department of Surgery, Institute of Clinical Sciences, Sahlgrenska Academy, Sahlgrenska University Hospital, University of Gothenburg, Per Dubbsgatan 15, 413 45 Gothenburg, Sweden

**Keywords:** Mechanism of injury, Rib fracture, Flail chest, Injury patterns, Operation, Chest wall trauma

## Abstract

**Purpose:**

Chest wall injuries are common in blunt trauma and associated with significant morbidity and mortality. The aim of this study was to determine the most common mechanisms of injury (MOI), injury patterns, and associated injuries in patients who undergo surgery for chest wall trauma.

**Methods:**

This was a retrospective study of trauma patients with multiple rib fractures and unstable thoracic cage injuries who were managed surgically at Sahlgrenska University Hospital during the period September 2010–September 2017. The MOI, injury severity score (ISS), new injury severity score (NISS), thoracic and associated injuries were recorded. Patients were categorized according to age (years): groups I (15‒44), II (45‒64) and III ( > 64). Unstable thoracic cage injuries were classified as sternal, anterior, lateral and posterior flail chest.

**Results:**

Two hundred and eleven trauma patients with a mean age (years) of 58.2 ± 15.6, mean ISS 23.6 ± 11.0, and mean NISS 34.1 ± 10.6 were included in the study. Traffic accidents were the most common MOI in Group I (62%) and falls in Group III (59%). The most common flail segments were lateral and posterior. Sternal and anterior flail segments were more common with bilateral injuries and traffic accidents, particularly frontal collisions. Injuries in at least three body regions were also more associated with traffic accidents. Diaphragmatic injury was seen in 18% of patients who underwent thoracotomy.

**Conclusions:**

The MOI associated with multiple rib fractures differs according to the age of the patient and is associated with different chest wall injury patterns and extra-thoracic injuries.

## Introduction

Thoracic trauma, encompassing trauma to the chest wall, lungs and cardiovascular system, accounts for approximately 23‒28% of trauma-associated mortality [1, 2]. Rib fractures are a common injury [3], especially in blunt trauma, and occur in approximately 40% of patients with thoracic trauma [4]. An increased number of rib fractures is associated with an increase in morbidity and there is a correlation between the number of ribs fractured, injury severity score (ISS) [5] and mortality [3, 6]. Multiple rib fractures are highly associated with pneumothorax, haemothorax and pulmonary injuries [4] and can lead to chest wall instability and flail chest [7] with subsequent respiratory insufficiency, a requirement for ventilator support and a high mortality [8]. Multiple rib fractures and flail chest are generally associated with high-energy trauma, such as road traffic accidents [4]. In the elderly, however, multiple rib fractures can occur after low-energy impact, such as falls that cause multiple, comminute and displaced rib fractures [9]. Elderly patients with rib fractures often present with a significantly less severe injury status and with lower ISS than younger patients, but have a higher rate of mortality [10, 11].

Rib fractures have previously been reported in 10% of trauma patients [3]. They are, however, frequently under-diagnosed because chest radiography fails to diagnose the majority of fractures [12, 13] and the true incidence may be underestimated. Computed tomography (CT) has a higher sensitivity for diagnosing rib fractures than radiography, with the added advantage of evaluating intra-thoracic injuries [13, 14]. Nevertheless, CT may also fail to diagnose rib fractures, particularly if these are non-displaced, chondral or anterior fractures [15].

Unstable thoracic cage injuries or flail chest occur in approximately 6% of patients with rib fractures [4] and have been described in 22% of patients with major chest trauma requiring intensive care [16], although the definition of major chest trauma was unclear in this study. The diagnosis of flail chest as anatomical or physiological can be disputed. Currently, and according to AIS 2008, the widely used definition is that flail chest is an anatomical diagnosis requiring three or more adjacent ribs fractured in more than one location [7]. This definition does not, however, take into account all injuries in the thoracic cage since sternal fractures in combination with rib fractures are not included in the definition. There is also lack of consensus as to what constitutes an anterior, lateral or posterior fracture, or an upper and lower rib fracture. These definitions are important because lower rib fractures are associated with increasing risk of intra-abdominal injuries [17, 18].

To our knowledge, no study has previously investigated an association between MOI and chest wall injury patterns. The aim of this study is to describe the MOIs, patterns of chest wall injuries, and associated injuries in patients who undergo open reduction internal fixation (ORIF) of chest wall injuries.

## Patients and methods

### Patients

#### Inclusion

We conducted a retrospective study of trauma patients (age ≥ 15 years) treated surgically for acute chest wall injuries at Sahlgrenska University Hospital during the period September 2010–September 2017. Those patients included in the study had been received directly at Sahlgrenska University Hospital or via another hospital in or outside of the region. Patients were identified in the surgical registry by a search using the ICD-10 codes GAD00, GAD03 and GAD96 for *thoracoplasty*, and GAE16, GAE23, GAE26, GAE30, GAE50 and GAE96 for *reconstruction of chest wall*.

#### Operative procedure

At our institution, the indications for emergency surgical stabilization of the chest wall are unstable thoracic cage injuries or flail chest, selected patients with multiple rib fractures and problems with pain control, visible chest wall deformity, lung herniation, and severe rib dislocation affecting internal organs. Open reduction internal fixation was performed using the MatrixRIB^®^ Fixation System (DePuy Synthes), consisting of pre-shaped angular locked plates and intra-medullary splints to stabilize rib fractures as described in previous studies [19, 20]. The operative technique varied, however, as some patients underwent thoracotomy with a non-muscle sparing approach, whereas others underwent a minimally invasive approach with or without thoracotomy.

### Methods

#### Patient demographics

Patient demographics, trauma demographics and operative procedures performed were collected from the patient files. Patients were categorized according to age (years): Group I (15‒44); Group II (45‒64); and Group III ( > 64). The MOI was divided into ten groups: (1) motor vehicle collision with other vehicle(s) (MVC other); (2) motor vehicle collision without other vehicle(s) (MVC single); (3) bicycle accident; (4) motorcycle accident; (5) pedestrian vehicle accident (PVA); (6) miscellaneous transport accidents (including gliding, skiing, jet-skiing, sledging, equine activities); (7) fall from the same level; (8) fall from height; (9) crush injury; and (10) assault by bodily force. The motor vehicle collision groups were further subdivided into three groups depending on whether a frontal or side collision had occurred or if this was unknown.

#### Injury diagnosis

Patients underwent a CT examination of the thorax with intravenous contrast medium often as part of a general, whole-body CT upon admission. The CT findings of intra-thoracic injuries were compared to operative findings in patients undergoing thoracotomy. Abbreviated Injury Scales (AIS) for the different body regions, Injury Severity Score (ISS) [5] and New Injury Severity Score (NISS) [21] were assessed. Injuries to the ribs, sternum, clavicle, scapula, lung, heart, diaphragm, and concomitant intracranial, abdominal, pelvic, spinal and extremity injuries were recorded.

#### Chest wall injuries

The number of fractured ribs, rib fractures, and the amount of fractures stabilized were studied. The rib cage was divided into sections by identifying the anterior axillary line (lateral border of pectoralis major muscle) and the posterior axillary line (anterior border of latissimus dorsi muscle) on cross-sectional CT images of the thorax at the level of the fourth rib. Fractures were classified as anterior (anterior to anterior axillary line), lateral (between anterior and posterior axillary lines), and posterior (posterior to posterior axillary line). The location of rib fractures was divided into zones: 1 (ribs 1‒4), 2 (ribs 5‒8), and 3 (ribs 9‒12). Chest wall deformity was determined on cross-sectional CT images. Fractures were defined as non-displaced or displaced and protruding if affecting internal organs. Flail chest was defined according to AIS criteria as fractures that involve three or more adjacent ribs fractured in more than one location [7]. In addition, flail segments were classified as sternal, anterior, lateral, and posterior. Sternal flail was defined as a segment consisting of at least two chondral or costal fractures bilaterally in conjunction with two horizontal sternal fractures or a vertical and horizontal fracture combined with two rib fractures on one side. Anterior flail was defined as a flail segment stretching between sternum and the anterior axillary line. Lateral flail was defined as a flail segment stretching from anterior ribs to posterior axillary line or anterior axillary line to posterior ribs. Posterior flail was defined as a segment stretching from posterior axillary line to the thoracic spine. Each patient could have more than one flail segment [7], although an individual rib fracture was not included in more than one flail segment.

### Statistical analyses

All statistical analyses were done using SPSS v21 software (IBM^®^ 2012). Results are shown as mean with standard deviation (SD) for continuous variables, and *n* and % for categorical variables. Continuous variables were compared using the independent samples two-tailed *T* test. Categorical variables were compared using the Pearson chi-square test and Fisher’s exact test. For variables without independent observations, Cochran’s *Q* test was used. Post hoc tests with subgroup analyzes were performed using *z* test. Significance was considered to be *p* < 0.05. For the comparison of MOI groups with categorical variables, the MOI groups were merged into three groups (fall, traffic accident and other accident) to avoid insufficient frequencies in each cell.

## Results

### Patient demographics

The study included 211 patients, 155 (73.5%) men and 56 (26.5%) women of mean age 58.2 ± 15.6 years who had been surgically managed for acute chest wall injuries during the 7-year study period. Sixty-five percent of patients had been received directly at Sahlgrenska University Hospital, 26% had been admitted via a regional hospital and 9% from a hospital outside the region. The main indications for surgery were flail chest (*n* = 184), multiple rib fractures and pain (*n* = 11), severely dislocated rib fractures affecting internal organs (*n* = 9), multiple rib fractures with lung herniation (*n* = 3), multiple rib fractures with chest wall deformity (*n* = 2), multiple rib fractures with massive haemothorax and diaphragmatic injury (*n* = 1) and multiple rib fractures with continuous air leakage (*n* = 1). One hundred and sixty-one (76%) patients underwent a thoracotomy in conjunction with chest wall stabilization (90 on the left side and 79 on the right side); eight of these patients underwent bilateral thoracotomies. There was no difference in the proportion of patients with unilateral injuries undergoing left vs. right side thoracotomy (*p* = 0.539).

### Mechanism of injury

Of the 211 patients included in the study, all but one had suffered blunt trauma. The exception was a patient who had been assaulted and stabbed in the chest, which caused severely dislocated rib fractures. Falls and traffic accidents were the most common MOI in patients undergoing surgical stabilization for chest wall injuries (Table 1). There was a significant difference in the proportion of patients suffering from falls, traffic or other accidents according to age group (*p* < 0.001). In Group I (15‒44 years), traffic accidents accounted for the majority (62%) of the trauma with the single, most common MOI being motorcycle accidents (29%). In the elderly, Group III ( > 64 years), the majority (59%) of injuries were due to falls of which 56% were from the same level. In contrast, falls and traffic accidents were equally distributed in Group II (45‒64 years). Subgroup analyses revealed that falls were more common in Group II and III than in Group I and traffic and other accidents were more common in Group I than in Group III (*p* < 0.05).

**Table 1:**
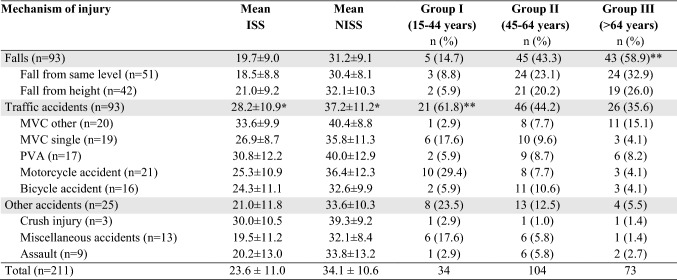
Injury severity scores according to mechanism of injury and mechanism of injury according to age groups in 211 patients surgically managed for acute chest wall injuries

*Highest ISS and NISS according to mechanism of injury using independent samples *T* test (*p* < 0.01)

**Most common mechanism of injury according to age group using subgroup analyses with *z* test (*p* < 0.05)

The mean ISS was 23.6 ± 11.0 and mean NISS was 34.1 ± 10.6 for patients included in the study. Injury severity scores were not equally distributed between the different MOI groups and mean ISS values were significantly higher in traffic accidents compared to falls (*p* < 0.001) and other accidents (*p* = 0.005) (Table 1). There was no difference in mean ISS between falls and other accidents (*p* = 0.526). Similarly, mean NISS was higher for traffic accidents compared to falls (*p* < 0.001), although there was no significant difference between mean NISS in other accidents compared to falls and traffic accidents (*p* = 0.260 and *p* = 0.152, respectively). The highest ISS and NISS values were seen in patients experiencing MVC with other vehicles, PVA and crush injury. There were no significant differences in either mean ISS or mean NISS between the subgroups of falls and other accidents. There were no significant differences in the distribution of age, MOI, and ISS and NISS values between men and women or the distribution of ISS and NISS values according to age groups.

### Chest wall injuries

Unilateral rib fractures were seen on the left and right sides in 80 and 64 patients, respectively. Sixty-seven patients had bilateral rib fractures. The mean numbers of fractured ribs and rib fractures in patients with unilateral injuries were 7.1 ± 2.1 and 12.2 ± 4.4, respectively. Fifty-seven percent of fractured ribs were operated and 44% of rib fractures were stabilized. There was no statistical difference in the number of fractured ribs (*p* = 0.930), rib fractures (*p* = 0.963) or percentages of ribs (*p* = 0.079) or fractures (*p* = 0.716) operated between patients with rib fractures on the left and right sides. Patients with bilateral injuries had significantly more fractured ribs (13.2 ± 4.0) and rib fractures (20.5 ± 7.5) compared to patients with unilateral injuries (*p* < 0.001). The percentage of ribs (37%) and fractures (32%) stabilized, however, was less than compared to unilateral injuries (*p* < 0.001). There was no significant difference in the number of fractured ribs (*p* = 0.509) or rib fractures (*p* = 0.860) between men and women.

**Fig. 1:**
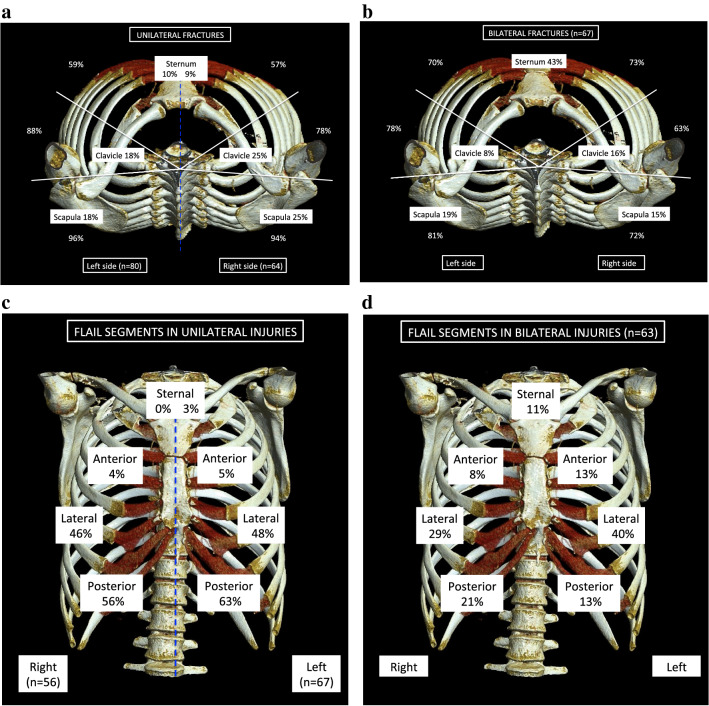
Percentage of anterior, lateral and posterior rib fractures, and fractures in sternum, clavicles, and scapulae in patients with **a** unilateral rib fractures on the left (*n* = 80) and right side (*n* = 64), respectively, and **b** bilateral rib fractures (*n* = 67). Percentage of sternal, anterior, lateral and posterior flail segments in **c** patients with unilateral rib fractures and flail chest on the left (*n* = 67) and right (*n* = 56) side, respectively, and **d** patients with bilateral rib fractures and flail chest (*n* = 63)

The incidence and location of fractures in ribs, sternum, clavicles, and scapulae in patients with unilateral injuries and bilateral injuries are shown in Fig. 1a, b. Comparison of proportions of sternal fractures between patients with unilateral (left vs. right side) and bilateral injuries with Pearson chi-square indicated a significant difference (*p* < 0.001) and subgroup analyses revealed significantly more sternal fractures in patients with bilateral injuries (*p* < 0.05). Clavicle and scapular fractures were equally distributed in patients with unilateral and bilateral injuries. Comparison of location of rib fractures with Cochran’s *Q* test showed posterior fractures being most common followed by lateral and lastly anterior rib fractures in patients with unilateral injuries (*p* < 0.001), whereas in patients with bilateral injuries, anterior, lateral and posterior rib fractures were equally common (*p* = 0.939). Anterior rib fractures were significantly more common in patients with bilateral injuries compared to patients with unilateral injuries (*p* < 0.001) (Table 2). Rib fractures were most common in zone 2 (99.5%), followed by zone 1 (81.5%) and zone 3 (76.8%) both in unilateral and bilateral injuries (*p* < 0.001). Zone 1 injuries were significantly more common in patients with bilateral than unilateral injuries (*p* < 0.001) and zone 3 rib fractures were more common in bilateral injuries than on the right side (*p* < 0.05).

**Table 2:**
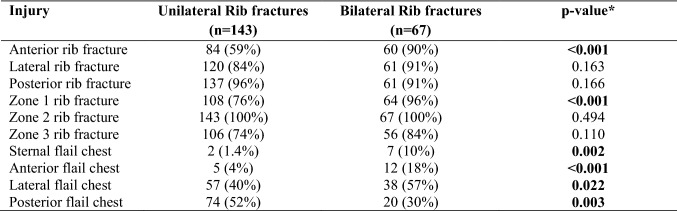
Comparison of chest wall injury patterns between unilateral and bilateral injuries in 210 trauma patients

Results from 210 of the 211 patients included in the study as data in one patient is missing

*Pearson’s chi square

Chest wall deformity was more common in patients with bilateral injuries (88%), compared to patients with unilateral injuries (65% left side vs. 59% right side) (*p* < 0.001). Ninety-nine percent of patients had at least one dislocated rib fracture and 40% percent had at least one protruding rib fracture. There was no significant difference in the occurrence of dislocation (*p* = 0.756) and protrusion (*p* = 0.388) between unilateral (left vs. right side) or bilateral injuries. Flail chest was present in 84% of patients with unilateral injuries on the left side, 88% with unilateral injuries on the right side and in 94% of patient with bilateral injuries (Fig. 1c, d). There was no significant difference in the proportion of flail chest between unilateral (left vs. right side) and bilateral injuries (*p* = 0.155). Ten patients had bilateral flail chest. Comparison of location of flail segments with Cochran’s *Q *test showed that lateral and posterior flail segments were most prevalent, followed by anterior and sternal flail segments (*p* < 0.001). For patients with bilateral injuries there were a significantly greater proportion of sternal flail chest (*p* = 0.002), anterior flail chest (*p* < 0.001) and lateral flail chest (*p* = 0.022) compared to patients with unilateral injuries (Table 2). Posterior flail chest was more common in patients with unilateral injuries than in patients with bilateral injuries (*p* = 0.003). Rib fractures in zone 1 were associated with anterior flail chest (*p* = 0.018) and lateral flail chest (*p* < 0.001). All patients with sternal flail chest had rib fractures in zones 1 and 2, whilst rib fractures in zone 3 were associated with posterior flail chest (*p* < 0.001). Despite radiological flail segments, clinical paradoxical breathing was only documented in 16% of patients with flail chest on the left side and 14% of patients with flail chest on the right side. In patients with bilateral injuries and flail chest, paradoxical breathing was recorded significantly more often than in unilateral injuries (43%, *p* < 0.001). There was a significant association between the reporting of paradoxical breathing and sternal and anterior flail segments (*p* < 0.05).

The association of MOI with chest wall injury pattern is shown in Table 3. The most common MOI in patients with unilateral injuries was falls, whereas the most common MOI in bilateral injuries was traffic accidents (*p* < 0.05). Rib fractures in zone 1 were more common in traffic accidents than falls, zone 2 rib fractures were equally common in all MOI groups and zone 3 rib fractures were more common in falls than traffic and other accidents (*p* < 0.05). Flail chest was equally prevalent in the three MOI groups (*p* = 1.000); however, all patients with PVA and crush injury had flail chest. Sternal and anterior flail segments were significantly more common in traffic accidents compared to falls and other accidents, while posterior flail chest was more common in falls than traffic and other accidents (*p* < 0.05). Patients in frontal collision MVC had more sternal and anterior flail segments than side collisions. While, posterior flail chest was more common in side collisions than frontal collisions. Lateral flail chest was equally common among the MOI groups and the most common type of flail in patients with PVA and motorcycle accidents (*p* < 0.001).

**Table 3:**
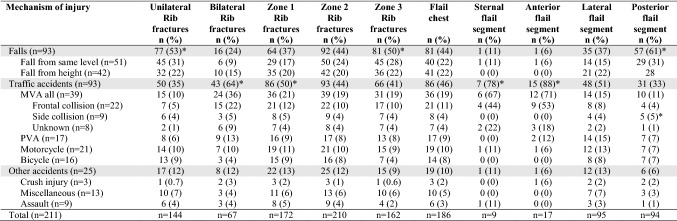
Chest wall injuries associated with mechanism of injury in 211 patients surgically managed for acute chest wall injuries

*Most common mechanism of injury with subgroup analyses (*p* < 0.05)

### Intra-thoracic injuries

**Fig. 2:**
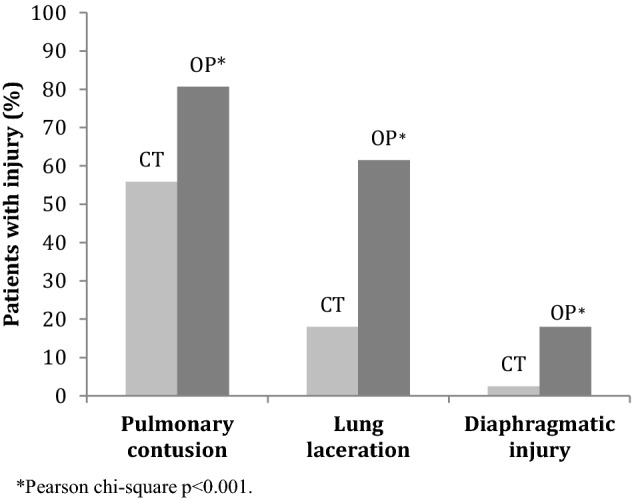
Percentage of patients with injuries seen on pre-operative CT and per-operatively in 161 patients undergoing thoracotomy in conjunction with chest wall stabilization

Intra-thoracic injuries were commonly found on pre-operative CT with 72% of patients with pneumothorax, 91% with haemothorax, 59% with pulmonary contusions, 16% with lung laceration, and 0.5% with bronchial injury. Cardiovascular injury occurred in 3.8% of patients. Diaphragmatic injury was diagnosed on CT in 1.9% of patients. Injuries that could be assessed per-operatively were found to be under-diagnosed on pre-operative CT. When comparing results in patients who subsequently underwent a thoracotomy (*n* = 161), 81% were found to have pulmonary contusions, 62% had lung laceration, and 18% had diaphragmatic injury (Fig. 2). The differences between CT and per-operative diagnoses were statistically significant (*p* < 0.001). The sensitivity of CT for diagnosing pulmonary contusions was 68%, lung lacerations 29% and diaphragmatic injuries 14% compared to operative diagnoses.

### Associated injuries

Isolated thoracic injury was found in only 12% of patients and was significantly more common in patients subjected to falls and other accidents than traffic accidents (*p* < 0.05) (Table 4). In contrast, injuries to at least three body regions were found in 64% of patients and were associated with traffic accidents to a significantly greater extent than falls and other accidents (*p* < 0.05). Traffic accidents were also associated with a significantly greater extent with abdominal solid organ injury, pelvic fracture, extremity fractures and spinal fracture than falls and other accidents (*p* < 0.05). Extremity fractures were particularly prevalent in PVA and found in 94% of patients. Motor vehicle collision with frontal collision was more commonly associated with injuries to multiple body regions (*p* < 0.001) compared to side collisions. Abdominal solid organ injury, pelvic fracture, extremity fracture and spinal fracture were more common in frontal collisions than side collisions (*p* < 0.05). Spinal fractures were seen in 96% of MVC in frontal collision. There was no significant difference in frequency of intracranial hemorrhage (*p* = 0.124), pulmonary contusions (*p* = 0.736), diaphragmatic injury (*p* = 0.628), and hollow viscus injury (*p* = 0.347) between the MOI groups.

**Table 4:**
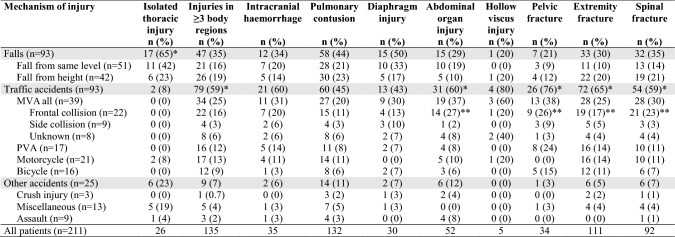
Frequency of injuries in different body regions according to mechanism of injury in 211 patients surgically managed for acute chest wall injuries

*Most common mechanism of injury with subgroup analyses (*p* < 0.05)

**Most common mechanism of impact when comparing MVA groups with subgroup analyses (*p* < 0.05)

## Discussion

In this retrospective study of 211 trauma patients who underwent ORIF due to acute chest wall injuries, we found that falls and traffic accidents were equally common and constituted the mechanism of injury in the majority (88%) of cases. This contradicts previous studies, which show that traffic accidents are a more common MOI in thoracic trauma [3, 4]. One possible explanation for the dissimilarity is the older age of the patients in this study (mean age 58 years). Another explanation may be the lower incidence of traffic accidents in Sweden compared to other countries [22]. We found an age-related variance with traffic accidents dominating the younger population compared to the older population where falls dominate. This does concur with previous studies that have shown an increased prevalence of fall accidents in older patients [23]. Previous studies suggest that elderly patients have lower ISS despite higher mortality [11, 3, 23]. Injury severity scores, ISS and NISS, were higher in traffic accidents, especially in MVC with other vehicle(s) and in PVA. We found that ISS was related to MOI rather than the age group of patients. Interestingly, there was no significant difference in ISS and NISS between falls from the same level and falls from height.

The indication for chest wall stabilization was in 87% of cases flail chest. Patients had at least one dislocated rib fracture. The majority of patients had unilateral rib fractures, although one-third had bilateral rib fractures. Bilateral rib fractures were more common in traffic accidents and associated with more rib fractures, although the proportion of fractures operated was less than when compared to unilateral injuries. More studies are needed to determine the optimal percentage of rib fractures to stabilize in flail chest, as stabilizing too few may not prevent deformity, and stabilizing too many may cause rigidity [24]. There was no difference in the number of rib fractures between men and women despite elderly women having a higher risk of osteoporosis [25]. Only 12% of patients had isolated thoracic trauma, which was associated with falls, whereas 64% of patients had injuries to at least three body regions, associated with traffic accidents. Traffic accidents were associated with abdominal organ injury, pelvic fracture, extremity fracture, and spinal fracture. In fact, 96% of patients in frontal collision MVC had spinal fractures and 94% in PVA had extremity fractures. Motor vehicle collision with frontal collisions was associated with a greater extent with injuries in at least three body regions and abdominal organ injury, pelvic fracture, extremity fracture and spinal fracture, than side collisions. Previous studies have also shown that collision type has a greater impact on injury severity than speed [26].

In our study, flail chest was based on anatomical criteria. We found signs of a “floating segment”, such as paradoxical breathing, in 25% of patients with flail chest. This was recorded in 43% of cases in patients with bilateral rib fractures and was associated with sternal and anterior flail segments. It is understandable that paradoxical breathing was more commonly seen in bilateral injuries as these have more frequent sternal and anterior flail segments, which are easier to discover. It is equally understandable that posterior flail segments do not produce a visible floating segment considering the overlying thick musculature. Whilst the occurrence of paradoxical breathing may have been underreported considering that this was a retrospective study, others have also reported a low prevalence of paradoxical breathing despite flail chest [27].

Patients had at least one rib fracture in zone 2, whereas zone 1 rib fractures were more common in patients with bilateral injuries and associated with anterior and lateral flail segments. Zone 3 rib fractures were associated with posterior flail segments. Other studies have similarly defined rib fractures as upper and lower or divided them into different zones than our study [28]. An international consensus as to how chest wall injury patterns should be defined would be beneficial. We developed the commonly used definition of flail chest to involve the sternum. We found lateral and posterior flail segments to be the most common injury patterns. Sternal fractures and anterior rib fractures were more common in patients with bilateral injuries, leading to sternal and anterior flail segments, whereas lateral and posterior rib fractures leading to posterior flail segments were more common in patients with unilateral injuries. We found an association between collision type and type of flail segment. In particular, sternal and anterior flail segments were found in frontal collision MVC, while posterior flail segments were found in side collision MVC. We had no information as to whether or not our MVC patients had been belted. Previous studies have shown a different injury pattern with a higher distribution of injuries in unbelted patients [29]. Flail chest was found in all patients with PVA and crush injury, included in the study. Lateral flail segments were the most common in PVA and motorcycle accidents, whereas posterior flail segments were more common in falls.

In our study, a high percentage (76%) of patients underwent thoracotomy. It is debatable as to whether or not thoracotomy or video-assisted thoracoscopic surgery (VATS) should be performed in conjunction with ORIF of rib fractures. The procedure allowed us to diagnose intra-thoracic injuries to a greater extent. We found that pre-operative CT failed to diagnose pulmonary contusions, lung lacerations, and diaphragmatic injuries in several patients. Ultrasound has been shown to be a valuable adjunct in the initial assessment of trauma patients [30] and is more sensitive than chest radiographs in diagnosing rib fractures [14], although the examination may be more time-consuming, be difficult in the presence of subcutaneous emphysema, be inaccessible to subscapular rib fractures and painful [31]. However, ultrasound can serve as a complement to CT with the added advantage of possible repetition without radiation, but it is still less sensitive than CT, despite having a high specificity concerning diagnosing intra-thoracic injuries [32]. At our hospital, ultrasound was used as a bedside tool in the resuscitation of our patients but we had not implemented a standardized protocol for assessing fractures in the sternum, cartilage or ribs, and therefore this modality was not used in our study. Also, pulmonary contusions often develop over time and it is possible that these could not be diagnosed on initial CT or ultrasound [33]. We found a low sensitivity for CT in diagnosing diaphragmatic injuries, which seem to be more prevalent than previously described. Zarour et al. reported a low incidence (0.9%) of traumatic diaphragmatic injuries, which mainly occurred in penetrating trauma [34]. There is a difference, however, between what has traditionally been described as traumatic diaphragmatic rupture secondary to blunt trauma and the diaphragmatic injuries seen in conjunction with rib fractures that more resemble lacerations after stab injuries. These are more common than previously known and we found them in 18% of patients undergoing thoracotomy in this study. It is important to diagnose diaphragmatic injuries as these can lead to late complications with high mortality [35, 36].

Even though CT was used in all our patients and is known to be the diagnostic tool of choice since chest X-rays fail to diagnose 75% of rib fractures seen on CT [13], we may have underestimated the number of rib fractures. Moreover, we have not described chondral injuries separately in this study. These can be difficult to diagnose and are frequently underestimated despite contributing to the development of sternal and anterior flail segments. Another limitation of our study is the variation of operative techniques since all patients did not undergo thoracotomy, and this may be a potential bias when assessing the number of patients with missed diaphragmatic injury.

The retrospective nature of the study is a limitation since a prospective study could have yielded additional information. The number of included patients was low when divided into the different mechanism of injury categories, which limited the comparative analyses. Notably, this is a study of patients undergoing ORIF and may not, therefore, be representative of all patients with multiple rib fractures and flail chest. Nevertheless, our study is a unique, in-depth analysis of the association of rib fractures and different types of chest wall injury patterns. It is important to recognize that patients with chest wall trauma have different injury patterns as this influences the operative approach in patients undergoing ORIF and may help raise suspicion for associated injuries.

## Conclusion

The MOI associated with multiple rib fractures and flail chest differs according to age group, and is associated with different chest wall injury patterns and risks of extra-thoracic injuries. Diaphragmatic injuries secondary to penetrating ribs are more common than previously thought. Traffic accidents and head-on collisions specifically are associated with polytrauma and severe injuries.
